# Simulating the Effect of Reinforcement Learning on Neuronal Synchrony and Periodicity in the Striatum

**DOI:** 10.3389/fncom.2016.00040

**Published:** 2016-04-29

**Authors:** Sébastien Hélie, Pierson J. Fleischer

**Affiliations:** Department of Psychological Sciences, Purdue UniversityWest Lafayette, IN, USA

**Keywords:** computational neuroscience, striatum, reinforcement learning, synchrony, periodicity

## Abstract

The study of rhythms and oscillations in the brain is gaining attention. While it is unclear exactly what the role of oscillation, synchrony, and rhythm is, it appears increasingly likely that synchrony is related to normal and abnormal brain states and possibly cognition. In this article, we explore the relationship between basal ganglia (BG) synchrony and reinforcement learning. We simulate a biologically-realistic model of the striatum initially proposed by Ponzi and Wickens (2010) and enhance the model by adding plastic cortico-BG synapses that can be modified using reinforcement learning. The effect of reinforcement learning on striatal rhythmic activity is then explored, and disrupted using simulated deep brain stimulation (DBS). The stimulator injects current in the brain structure to which it is attached, which affects neuronal synchrony. The results show that training the model without DBS yields a high accuracy in the learning task and reduced the number of active neurons in the striatum, along with an increased firing periodicity and a decreased firing synchrony between neurons in the same assembly. In addition, a spectral decomposition shows a stronger signal for correct trials than incorrect trials in high frequency bands. If the DBS is ON during the training phase, but not the test phase, the amount of learning in the model is reduced, along with firing periodicity. Similar to when the DBS is OFF, spectral decomposition shows a stronger signal for correct trials than for incorrect trials in high frequency domains, but this phenoemenon happens in higher frequency bands than when the DBS is OFF. Synchrony between the neurons is not affected. Finally, the results show that turning the DBS ON at test increases both firing periodicity and striatal synchrony, and spectral decomposition of the signal show that neural activity synchronizes with the DBS fundamental frequency (and its harmonics). Turning the DBS ON during the test phase results in chance performance regardless of whether the DBS was ON or OFF during training. We conclude that reinforcement learning is related to firing periodicity, and a stronger signal for correct trials when compared to incorrect trials in high frequency bands.

## Introduction

The study of rhythms and oscillations in the brain is gaining attention (Wang, 2010). While it is unclear exactly what the role of oscillation, synchrony, and rhythm is (Walters and Bergstrom, 2010), it appears increasingly likely that synchrony is related to normal and abnormal brain states and possibly cognition. For example, impaired beta- and gamma-band synchronization (16–31 and 32–100 Hz, respectively) has been observed in schizophrenic patients performing a number of perceptual tasks (for a review, see Uhlhaas and Singer, 2010). These are thought to reflect impairment in long-range cortical connectivity (Wang, 2010). As another example, people suffering from Parkinson's disease show abnormal synchrony in the beta range in the basal ganglia (BG), especially in the globus pallidus and the subthalamic nucleus (Bergman et al., 2010). Parkinson's disease is characterized by the accelerated death of dopamine (DA) producing neurons (Hélie et al., 2012), and there is evidence showing that the death of DA-producing neurons increases neuronal synchrony (Walters and Bergstrom, 2010). Treatment with L-DOPA tends to restore normal rhythmic activity in the BG (Wichmann and DeLong, 2010).

Dopamine also plays an important role in many cognitive functions (e.g., Nieoullon, 2002). One of the most well-established roles of DA in cognitive function is related to feedback-driven (instrumental) learning (Schultz et al., 1997; Ashby and Helie, 2011). Specifically, DA levels in the BG increase when an unexpected reward is received, dip when an expected reward fails to appear, and change with a high-enough temporal resolution to serve as a reward signal in reinforcement learning (Helie et al., 2015). Perhaps relatedly, people suffering from Parkinson's disease, who have overall lower DA availability, suffer from many cognitive deficits related to memory, learning, visuospatial skills, and attention (Price et al., 2009).

In this article, we explore the relationship between BG rhythm and reinforcement learning. The evidence reviewed above suggests that both synchrony and learning are related to DA levels, but it is unclear if synchrony and learning are directly related. Specifically, we simulate a biologically-realistic model of the striatum initially proposed by Ponzi and Wickens (2010) and enhance the model by adding plastic cortico-BG synapses that can be modified using reinforcement learning (Ashby and Helie, 2011). The effect of reinforcement learning on striatal rhythmic activity is then explored, and disrupted using simulated deep brain stimulation (DBS) (Rubin and Terman, 2004). Deep brain stimulation uses a brain implant to treat brain dysfunctions such as Parkinson's disease, dystonia, and depression (among others, see Wichmann and DeLong, 2010). The stimulator injects current in the brain structure to which it is attached, which affects neuronal synchrony. To anticipate, training the model without DBS yields a high accuracy in the learning task, along with an increased firing periodicity (for individual neurons) and a decreased firing synchrony between neurons in the same assembly. In addition, a spectral decomposition shows a stronger signal for correct trials than incorrect trials in high frequency bands. If the DBS is ON during the training phase, but not the test phase, the amount of learning (i.e., improvement in accuracy) in the model is reduced, along with firing periodicity. Similar to when the DBS is OFF, spectral decomposition shows a stronger signal for correct trials than for incorrect trials in high frequency domains, but this phenoemenon happens at higher frequency bands than when the DBS is always absent. Synchrony between the neurons is not affected. Finally, the results show that turning the DBS ON at test increases both firing periodicity and striatal synchrony, and spectral decomposition of the signal shows that neural activity synchronizes with the DBS fundamental frequency (and its harmonics). Perhaps not surprinsingly, turning the DBS ON during the test phase results in chance performance regardless of whether the DBS was ON or OFF during training. We conclude that reinforcement learning is related to firing periodicity, and a stronger signal for correct trials when compared to incorrect trials in high frequency bands.

## Materials and methods

### Model

Cognitive computational neuroscience models (Ashby and Helie, 2011) of the BG typically represent the striatum as a fully interconnected winner-take-all network of neurons (for a review, see Helie et al., 2013). While the winner-take-all dynamic is well-supported by the segregated channels and the striatum's association with the selection of mutually exclusive actions, the striatum is not as fully interconnected as these models imply. Striatal interconnection is sparse (Plenz and Wickens, 2010). One model that accurately simulates this connectivity is the Ponzi and Wickens model (2010). The Ponzi and Wickens model includes a single layer of neurons with random sparse inhibitory interconnectivity, and the model has been shown to automatically produce clusters of neurons (cell assemblies) that fire in synchrony (as in a real striatum) under random input conditions. Accurately modeling sparse striatal connectivity is essential to accurately modeling BG synchrony, so the Ponzi and Wickens model was used as a starting point to explore the possible relationship between BG synchrony and learning. All the equations used to implement the Ponzi and Wickens model are shown in the Supplementary Material. The proposed expansion of the model is described in the remainder of this section.

### Cortical input

The first addition made to the Ponzi and Wickens model is a cortical input grid of 10,000 input nodes. We use the term “nodes” to emphasize that we did not model membrane potentials or spiking behaviors in these units. The cortical nodes were directly activated by the external stimuli (modeled as boxcar functions) and filtered through Gaussian functions. To facilitate this, the cortical nodes were conceptually organized into a 100 × 100 grid with a node at each vertex. The output of cortical node C_ij_ is calculated using a bivariate normal function with peak at (*x, y*) and a standard deviation of 8.5 (and no covariance). Stimuli had two dimensions which were defined as coordinates: *a* and *b*. Both *a* and *b* fall within the range of 0 to 100. The amplitude of the normal distribution is scaled so that if the coordinates of a stimulus, *a* and *b*, are equal to *i* and *j*, respectively, then C_ij_'s output is 1.0.

Connections between the cortical layer and the striatal layer were randomly determined with the restriction that each striatal neuron received input from exactly 5000 cortical nodes. This matches the number of cortical inputs to a striatal neuron in the rat striatum (Wickens and Arbuthnott, 2010). The strength ω_*C*_*ij*__*S*_*k*_ of the connection between a cortical node C_ij_ and striatal neuron S_*k*_ is initialized using a normally distributed random variable with mean μ_*k*_ and standard deviation 0.35, the result of which is divided by the expected number of cortical nodes with output greater than 0.5 (i.e., the area of the cross section of the Gaussian at amplitude 0.5. μ_k_ is a normally distributed random variable associated with S_k_ with mean 3.5 and standard deviation 0.35). This allows the total input received by a striatal neuron to fall in the same range as in Ponzi and Wickens (2010).

### Learning

There is evidence suggesting that striatal dopamine acts as a reward signal (Schultz et al., 1997). Unexpected reward causes dopamine levels to rise above baseline and unexpected punishment or a failure to receive an expected reward causes dopamine to fall below baseline. In the model the expected reward for trial *n*+*1* is defined as (Ashby and Helie, 2011):

$$P_{n+1}=P_{n}+.075(R_{n}-P_{n})$$

with *R*_*n*_ being the received reward at trial *n*. If the model responded correctly *R*_*n*_ = 1, otherwise *R*_*n*_ = −1. The dopamine level after feedback is received is:

$$D_{n}=\{\begin{array}{cc} 1 & if\left(R_{n}-P_{n}\right)>1\\ 0 & if\left(R_{n}-P_{n}\right)<-0.25\\ 0.8\left(R_{n}-P_{n}\right)+0.2 & else \end{array}$$

where 0.2 is the baseline level of dopamine.

The weight of each cortico-striatal connection changes as a function of the level of dopamine *D*_*n*_, the output of the pre-synaptic (cortical) node *C*_*A*_*(t)*, and the excitation of the post-synaptic (striatal) neuron *V*_*B*_*(t)*:

$$\begin{array}{l} \omega_{A,B}\left(n+1\right)=\omega_{A,B}\left(n\right)\\ \;+\alpha_{\omega }\int C_{A}\left(t\right)\;dt{\left[\int {\left[V_{B}\left(t\right)\right]}^{+}dt-\theta_{NMDA}\right]}^{+}\\ \;{\left[D_{n}-D_{base}\right]}^{+}\left[\omega_{max}-\omega_{A,B}\left(n\right)\right]\\ \;-\beta_{\omega }\int C_{A}\left(t\right)\;dt{\left[\int {\left[V_{B}\left(t\right)\right]}^{+}dt-\theta_{NMDA}\right]}^{+}\\ \;{\left[\;D_{base}-D_{n}\right]}^{+}\omega_{A,B}\left(n\right)\\ \;-\gamma_{\omega }\int C_{A}\left(t\right)\;dt{\left[\theta_{NMDA}-\int {\left[V_{B}\left(t\right)\right]}^{+}dt\right]}^{+}\\ \;{\left[\int {\left[V_{B}\left(t\right)\right]}^{+}dt-\theta_{AMPA}\right]}^{+}\omega_{A,B}\left(n\right) \end{array}$$

where ω_*max*_ = 16, α_ω_ = 7 × 10^−11^, β_ω_ = 2 × 10^−11^, γ_ω_ = 5 × 10^−12^, and *[X]*^+^ = *X* if *X* > *0* and *[X]*^+^ = 0 otherwise. Each term represents a different case. (1) If the level of dopamine *D*_*n*_ is greater than the baseline *D*_*base*_ and the sum of the positive membrane potential of the striatal neuron is high enough to activate the NMDA receptors (i.e., $\int {\left[V_{B}\left(t\right)\right]}^{+}dt$ is above the threshold θ_*NMDA*_ = 25), the connection weight is increased (long-term potentiation). (2) If the level of dopamine is *below* baseline and the sum of the positive membrane potential of the striatal neuron is again high enough to activate the NMDA receptors, the connection weight is decreased (long-term depression). (3) Finally, if the sum of the positive membrane potential of the striatal neuron is not high enough to activate the NMDA receptors but high enough to activate AMPA receptors (i.e., $\int {\left[V_{B}\left(t\right)\right]}^{+}dt$ is above the threshold θ_*AMPA*_ = 10), the connection weight is decreased. If the positive membrane potential of the striatal neuron is not sufficient to activate AMPA receptors, no change is made. Note that only the positive postsynaptic membrane potential is integrated because at resting membrane potentials, an extracellular Mg2+ plug blocks the mechanisms of long-term potentiation/depression. More biological details justifying the learning equation are reviewed in Ashby and Helie (2011).

The synaptic plasticity modeled by the learning equation has been verified (for reviews see, e.g., Arbuthnott et al., 2000; Wickens et al., 2003) and previously used to model instrumental learning (e.g., Ashby and Helie, 2011; Hélie et al., 2012). However, the mechanism underlying plasticity at inhibitory synapses (e.g., GABA) is still unclear (Castillo et al., 2011), and the lateral inhibition in the Ponzi and Wickens model is responsible for the rhythmic activity in the simulated striatum. The main goal of this article is to study how reinforcement learning affects the rhythmic activity in the striatum. Hence, the possible plasticity of the inhibitory lateral connections within the striatum was not modeled, and inhibitory lateral connections were fixed in all the simulations included in this article.

### Deep brain stimulation

The goal of the DBS manipulation in this article is to disrupt periodicity and striatal synchrony and observe whether learning is affected. It is well-known that inputting a correlated signal in a network increases synchrony (Wang, 2010). Thus, we used Rubin and Terman's (2004) model of DBS to increase periodicity and synchrony in the new model. Rubin and Terman's original model is described by:

$$I_{DBS}\left(t\right)=i_{D}H\left(sin\left(\frac{2\pi t}{\rho_{D}}\right)\right)\left(1-H\left(sin\left(\frac{2\pi (t+\delta_{D}}{\rho_{D}}\right)\right)\right)$$

where *i*_*D*_ = 200 is the amplitude of the DBS input, ρ_*D*_ = 6 ms is the period of the input, δ_*D*_ = 0.6 ms is the duration of the input, and H() is the Heaviside function (i.e., it returns 0 for negative arguments and 1 for positive arguments). To put it simply this model of a DBS adds a square signal of amplitude *i*_*D*_ for δ_*D*_ ms every period of ρ_*D*_ ms. At each time step, I_*DBS*_ is calculated and added to the input of every striatal neuron. While the above equation is a realistic model of a DBS, it increases the total activity in the network, which is unwanted for the present purpose. We added an additional term to the DBS model to keep the total input received by each striatal neuron the same when the DBS is ON or OFF. Since the intent is to manipulate the periodicity and synchrony of the network with as few side effects as possible, the DBS model was modified as follows:

$$\begin{array}{l} I_{DBS}\left(t\right)=i_{D}H\left(sin\left(\frac{2\pi t}{\rho_{D}}\right)\right)\left(1-H\left(sin\left(\frac{2\pi (t+\delta_{D}}{\rho_{D}}\right)\right)\right)\\ \;=-\frac{\delta_{D}i_{D}}{\rho_{D}} \end{array}$$

As a result, over the duration of one period the net sum input of the DBS model used in the present work is 0. This deviates from DBS that are implanted in real brains but better serves the purpose of modifying rhythmic activity without increasing the total input. The input from the DBS at time *t* [*I*_*DBS*_*(t)*] is added to the input of all neurons.

### Simulation method

The first step in the simulation was to generate random striatal maps composed of 100 neurons and identify clusters. We used the same methodology as Ponzi and Wickens (2010), but changed the clustering algorithm for the rate time series from *k*-means to the gap statistic from Tibshirani et al. (2001). The main advantage of using the gap statistic is that this measure will not only cluster the data but also find the number of clusters best suggested by the data, including the possibility of a single cluster in the case of undifferentiated data. The gap statistic clusters the data multiple times using *k*-means clustering for *k* = 1, 2, …, *k*_*max*_ where *k*_*max*_ is the maximum number of clusters selected by the experimenter. For each resulting clustering, the pooled within-cluster sum of squares around the cluster means W_k_ is calculated. Log(W_k_) is then compared to its expectation under an appropriate unclustered distribution of the data. The value of *k* for which log(W_k_) falls farthest below its expectation is the appropriate *k* to use for *k*-means clustering (for details, see Tibshirani et al., 2001). Using the gap statistic ensured that we used a striatal network that naturally generated two clusters of neurons. Twenty different striatal networks were randomly generated and the network that resulted in the most consistent clustering (i.e., the neurons were consistently clustered in the same pattern across multiple trials with randomly fluctuating input) was selected. In this case, the chosen network presented two clusters of size 40 and 60 (respectively). This set of striatal connections and the resulting clustering were used in all the following simulations.

The model was trained on a simple categorization task typical of human cognitive research (e.g., Ashby and Gott, 1988). In a categorization task, one stimulus is presented to the participant in each trial, and the participant needs to assign the stimulus to one of a number of categories. After a response is made, the participant receives accuracy feedback, and a new trial is initiated. At the beginning of the task, the participant does not know the stimulus—response associations and responds randomly, but accuracy improves as the participant receives feedback (instrumental learning). In the present simulation, a 2D stimulus was uniformly sampled from the 0…100 space at the beginning of each trial. The input space was sectioned into two catagories to match the two groups of striatal neurons (cell assemblies) found through clustering. In all trials, an input stimulus with an X-coordinate < 50 was a member of category A and an input with X-coordinate ≥50 was a member of category B. Category membership was not dependent on the value on the Y-coordinate. The cortical input signal was filtered through Gaussian functions as described in Section “Cortical Input”.

For each trial the model was run for 3000 ms after which the average total positive activation per neuron was calculated for each cluster. If Cluster A's total was higher the model was treated as having categorized the stimulus as part of category A. Otherwise the model categorized the stimulus as belonging to category B. In this article a *trial* refers to running the model for 3000 ms with a single stimulus. During the training phase, feedback was provided to the model (as a DA increase or dip) at the end of each trial and the learning equation was applied to each cortico-striatal weight. Note that the inhibitory striatal weights were constant throughout the simulation. The test phases were identical to the training phase, except that learning was disabled to observe a snapshot of the model performance in a constant state. When learning is disabled the DA level is not calculated, the cortico-striatal connection weights are not adjusted and the reward prediction is not updated. A *test block* is a series of trials with constant cortical-striatal connections.

A *simulation* went as follows: First, the cortico-striatal connections are initialized as described in Section “Cortical Input”. Second, there is a test block (pretest) in which learning was disabled. The pretest was used to gather 40 data points under each of the following 4 conditions: model ran with the DBS ON (designated “Test+”) and correctly responded (designated “C”), model ran with the DBS ON and incorrectly responded (designated “F”), model ran with the DBS OFF (designated “Test−”) and correctly responded, and model ran with the DBS OFF and incorrectly responded. Next, learning was enabled and there were 2000 training trials for the network to learn the stimulus—response associations. Training was run under one of two conditions. Either the DBS was turned ON for all training trials (designated “Learn+”) or it was turned OFF for all training trials (designated “Learn−”). Fourth, learning was again disabled and another test block (post-test) was conducted where 40 more results are gathered under each condition as described in the pretest above. One hundred simulations were run with the DBS ON at training and another 100 simulations were run with the DBS OFF at training.

In addition to the main simulation described above, 23 additional simulations were run in the Learn-/Test- condition (i.e., no DBS) with a test block conducted after every 100 training trials. These were used to compute the correlation between performance and periodicity as learning developed (more later).

### Data analysis

**Figure 1:**
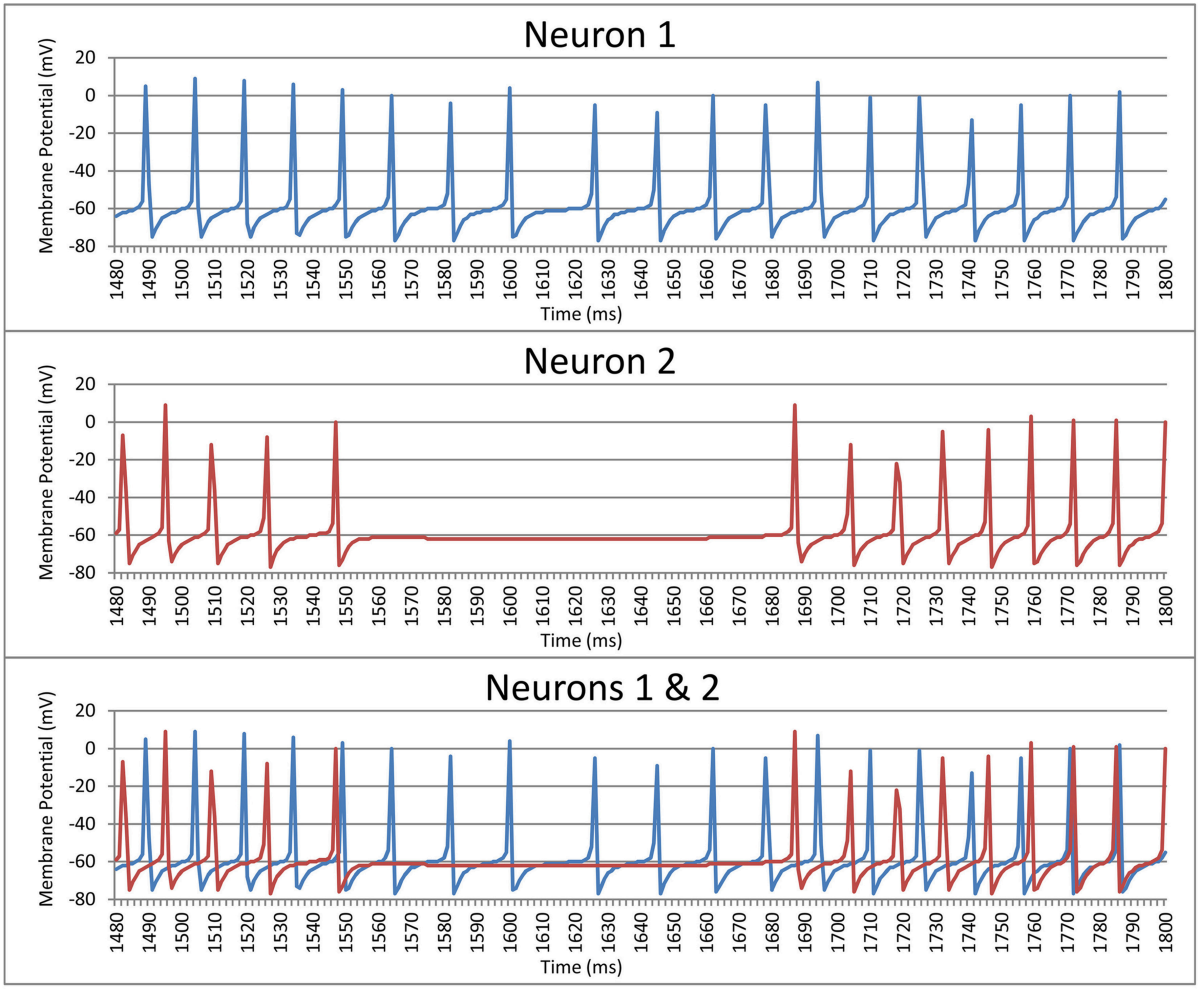
**Spike trains for two striatal neurons over a 320 ms time window prior to training the model**.

We used the Synchronization Index (SI), also called Vector Strength (VS), described in Kuebler and Thivierge (2014) to evaluate the neurons' spiking in two ways. First we used SI as written

(1) $$\begin{array}{l} VS=\frac{1}{N}\sqrt{{\left(\sum_{i=1}^{N}cos(\theta_{i})\right)}^{2}+{\left(\sum_{i=1}^{N}\sin(\theta_{i})\right)}^{2}}\\ \;\theta_{i}=2\pi \frac{mod\left(\frac{t_{i}}{c}\right)}{c} \end{array}$$

where *t*_*i*_ is the spike time of the *i*th spike and *c* is the period of a sine wave. We used this as a measurement of the periodicity of the neuron. The period was chosen to be equal to the mode of the intervals (rounded to the nearest ms) between spikes of that neuron. If the second mode was 1 ms off from the first mode the period *c* was instead set to the mean of the two modes weighted by their frequency. Put simply, periodicity is a measure of the synchrony between each neuron and a regular sine-wave function with a phase duration equal to the most common inter-spike delay for this neuron. For example, Neuron 1 in Figure 1 shows a neurons where the mode of the interspike delay is 15.3 ms, so this neuron's periodicity is calculated by computing its synchrony with a sine-wave function with a period of 15.3 ms. The periodicity of Neuron 1 is 0.249. Likewise, the mode of the interspike delay for Neuron 2 in Figure 1 is 13.5 ms, and its periodicity is 0.293.

The second measure compared the synchrony between the spike times of two neurons. Each neuron is paired with every other neuron in its cluster (cell assembly). Each neuron in a pair acts as a reference to the other neuron in that pair. For each *t*_*i*_ the period *c* is the length of the spike interval of the reference neuron that *t*_*i*_ falls within. What this means is that, for example, if the neuron currently being evaluated spiked halfway through each interval between the spikes of the reference neuron then the neuron being evaluated would be judged to be perfectly synchronized with the reference neuron (i.e., phase is not considered). For example, Neuron 1 in Figure 1 has a synchrony of 0.210 with Neuron 2, and Neuron 2 has a synchrony of 0.228 with Neuron 1. It should be noted that these measures of periodicity and synchrony (Equation 1) only give meaningful results if the neuron(s) being examined spike(s) more than once. Neurons spiking more than once are referred as *active neurons*.

## Results

Each trial of the model was run for 3000 ms (see Section “Simulation Method” above). For each trial, the first second was considered a burn-in period used to stabilize the model and discarded. All the following results focus on the last 2 s of each trial (stable period).

### Model without DBS

**Figure 2:**
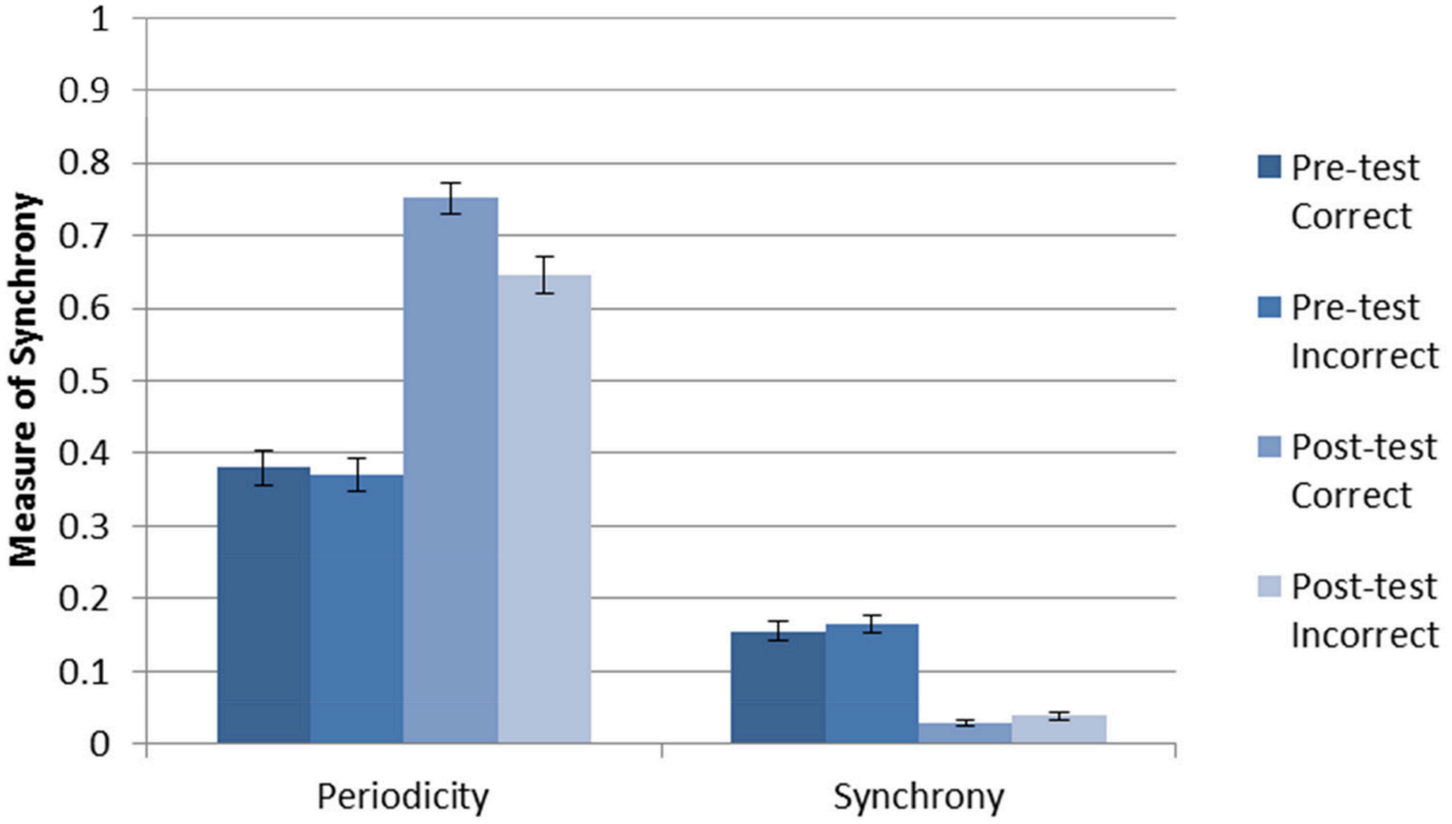
**Periodicity and synchrony of the model without DBS (Learn-/Test-) calculated using Equation (1)**. There is an increase in periodicity after training, especially for correct trials.There is a decrease in striatal synchrony from pre-test to post-test, and this effect is similar for correct and incorrect trials.

The average accuracy without DBS after 2000 trials of training was 0.911 with a standard deviation 0.097. The (within-neuron) periodicity and (between-neuron) striatal synchrony was separately calculated for each trial type (pre-test C, pre-test F, post-test C, post-test F) as follows. For each trial, periodicity was calculated individually for each active neuron, and synchrony was calculated for each pair of active neurons within each cluster (cell assembly). The mean periodicity and synchrony was then calculated for each trial. Next, trial-averaged synchronies and periodicities were calculated for each of the four trial types. Figure 2 shows the results for the Learn-/Test- condition. The error bars are calculated from the trial-level data. As can be seen, there was an increase in periodicity after training (post-test). In addition, posttest periodicity was higher for correct trials than for incorrect trials. These results suggest that neurons fire more regularly (i.e., higher periodicity) as the network becomes more accurate after being trained in the absence of DBS.

The results for striatal synchrony are also shown in Figure 2. As can be seen, training decreased striatal synchrony. This suggests that training with reinforcement learning tends to desynchronize the firing of striatal neurons within a cell assembly. Unlike the increase in periodicity, this decrease in striatal synchrony is non-specific and does not appear to be related to the accuracy of the response.

One possible explanation for the increased periodicity is that fewer neurons in the network were active after training, and thus there were fewer active neurons sending inhibition. Indeed, training did increase the number of neurons that did not fire any spikes. The mean number of active neurons (i.e., neurons that fired) during pre-test trials was 20.2 with a standard deviation of 14.04. In post-test trials the mean number of active neurons was reduced to 11.86 with a standard deviation of 3.46. Further, the probability that one of the active neurons inhibits another active neuron in pre-test trials is 0.10 while in post-test trials that probability is 0.05. This increased the average firing rate of active neurons from 32 to 129 Hz. This change in firing rate could account for the observed change in neural synchrony. Indeed, randomly generating spike times at the pre- and post-test average spike rates produced synchrony in the same range as those observed pre- and post-test in the model (respectively). However, this changes in the firing rates was not responsible for the observed changes in periodicity because generating random spike times at the firing rates observed for pre- and post-test always yielded levels of periodicity similar to those observed in the pre-test phase of the simulation.

**Figure 3:**
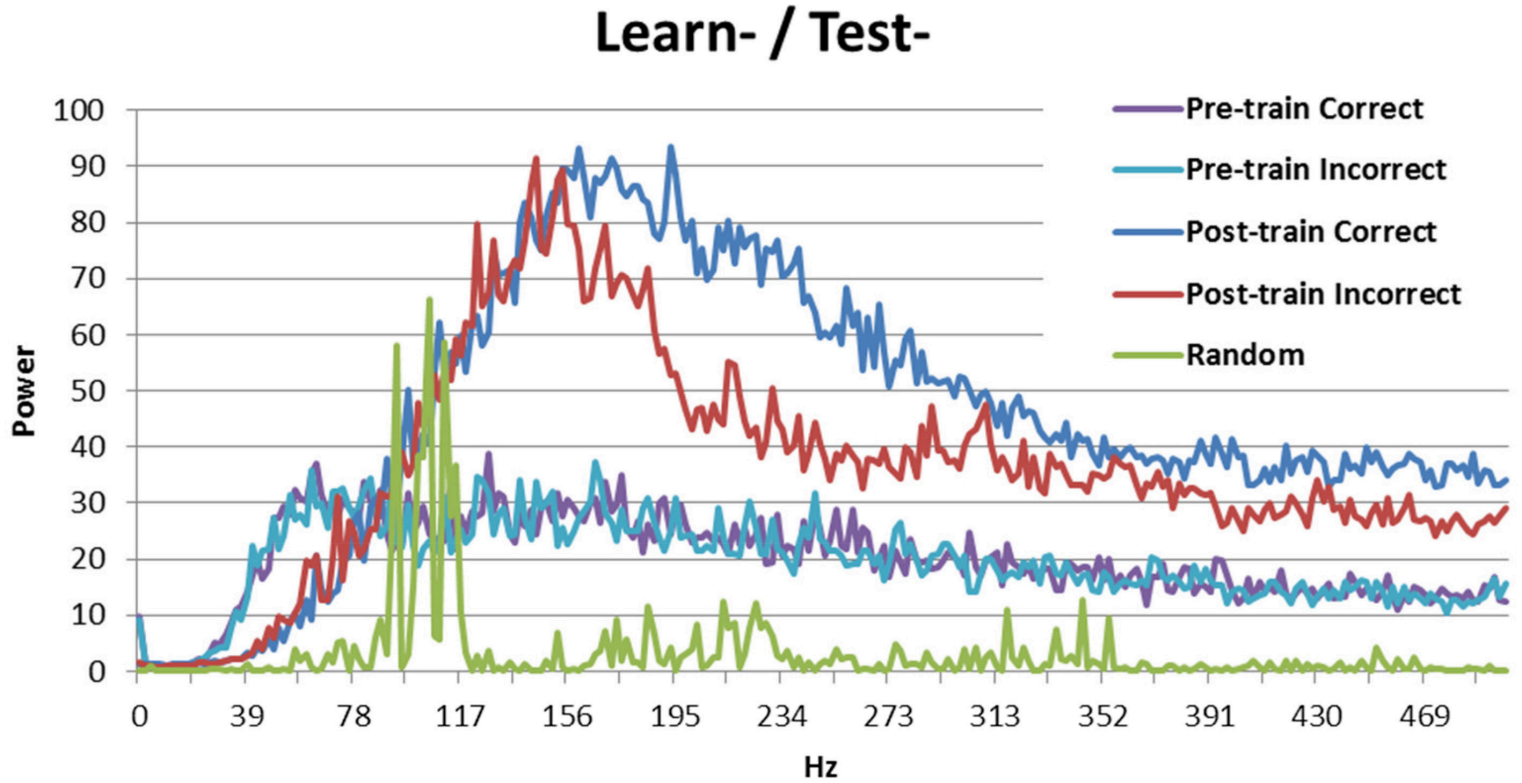
**Power spectrum of the model without DBS (Learn-/Test-)**. Before training correct and incorrect trials are indistinguishable. After training correct trials have a stronger signal than incorrect trials in high frequency bands. The power spectrum of the Ponzi and Wickens model under random input condition is also included. In the random condition, input varies across cells and is drawn from a uniform distribution over the interval (4.51, 5.51). The input is redrawn every 10 ms.

Breaking down the periodicity into component frequencies using the Fourier transform (Figure 3) shows that the signal for random activation (as used by Ponzi and Wickens, 2010) is mostly located in the gamma frequency range (as expected, green line). Activation from the simulated cortex added in this research increases the frequency of the signal. Correct and incorrect trials are indistinguishable before training (purple and teal), but the signal is stronger in high frequency bands for correct trials (blue) than for incorrect trials (red) after training. This result is consistent with those obtained by Brincat and Miller (2015), who measured synchrony between the prefrontal cortex and hippocampus in monkeys. Brincat and Miller argued that the stronger component in high frequencies for correct responses may correspond to long-term potentiation, whereas the weaker component in high frequency for incorrect responses may correspond to long-term depression (in line with spike timing dependent plasticity: Dan and Poo, 2004). In the present simulation, correct trials lead to long-term potentiation while incorrect trials lead to long-term depression, which supports Brincat and Miller's explanation.

### Model with DBS

The previous simulations show that (1) the model can learn the task, (2) that training increases periodicity, especially for correct trials, (3) training unspecifically reduces striatal synchrony (4) training increases the firing rate of active neurons, which can account for (3) but not (2) and, (5) after training, correct trials show a stronger component in high frequency bands than incorrect trials. This subsection explores the effect of turning ON the DBS during training only, during test only (both pre- and post-test), or during both. The dependent variables are the same as in Section “Model Without DBS”, namely accuracy, periodicity, striatal synchrony, and power spectrum.

**Figure 4:**
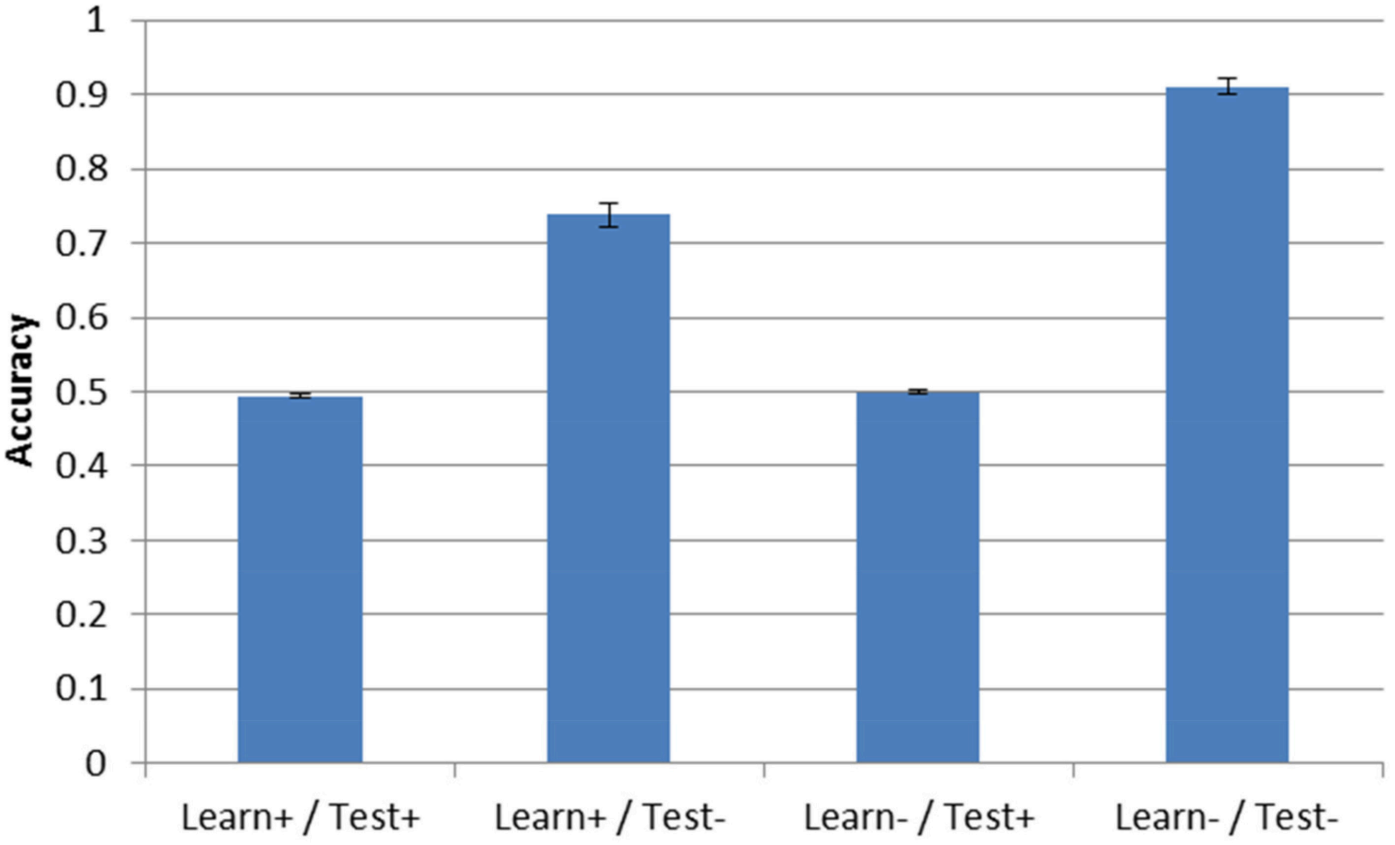
**Model accuracy after training for the three DBS conditions**. Accuracy without DBS (Learn-/Test-, from Section “Model Without DBS”) is also included for comparison. Performance is near-chance (0.5) when the DBS is turned ON at test.

Post-test accuracy for the three simulation conditions with DBS ON is shown in Figure 4. Note that accuracy in the Learn-/Test- corresponds to the condition described in Section “Model Without DBS” above and is shown in all the following figures to facilitate comparison. As can be seen, the presence of DBS at test is enough to reduce accuracy to chance performance (0.5). This is true even if the model was trained under the same conditions that is was tested on (e.g., Learn+/Test+). However, when DBS is turned ON during training but not during testing (Learn+/Test−), accuracy is 73.8%, which is well-above chance performance but still substantially less than when DBS is not turned ON during either the test or training phase (Learn-/Test-, accuracy = 91.1%).

**Figure 5:**
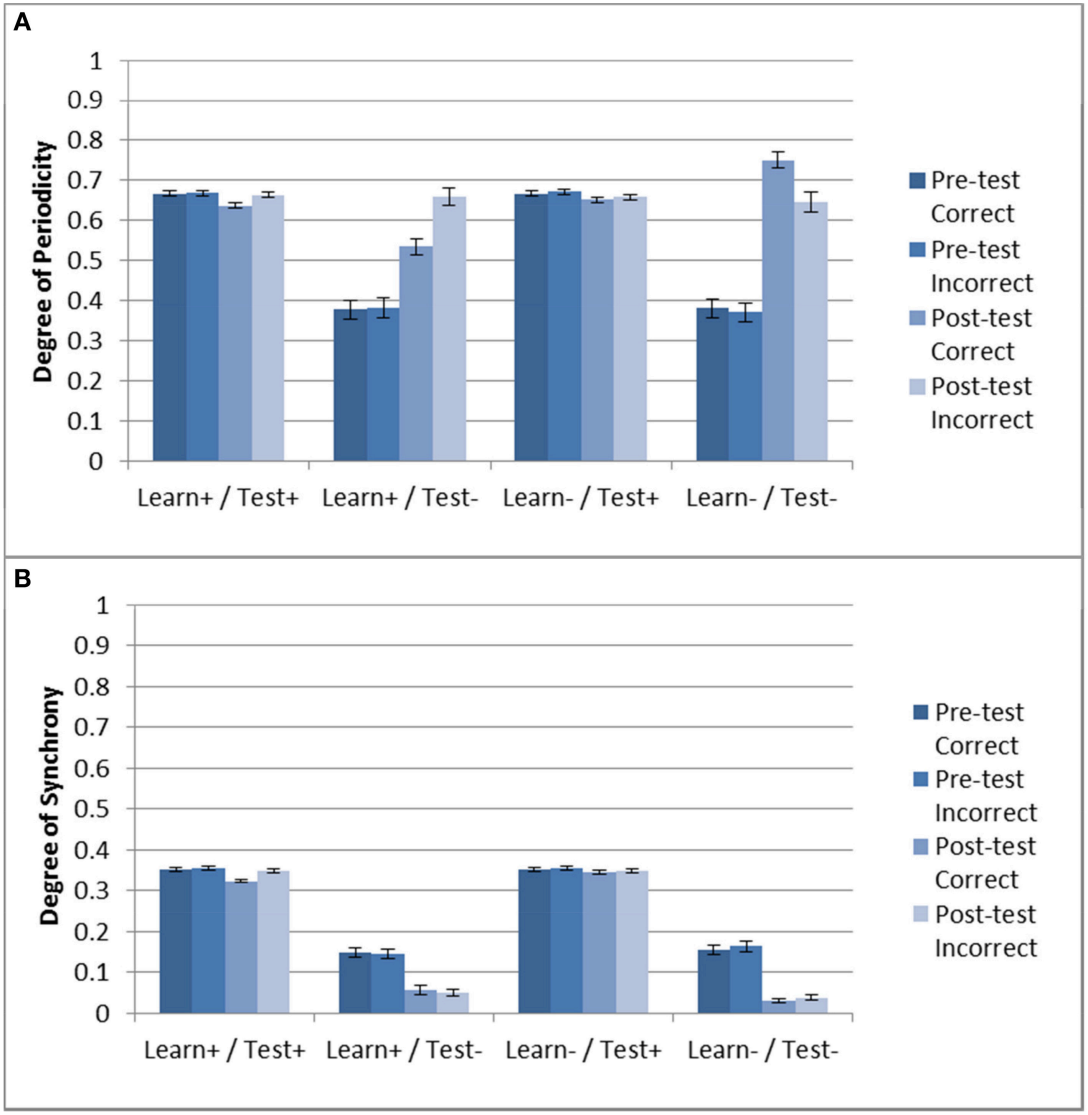
**Periodicity and striatal synchrony (A,B) under the three DBS conditions**. The Learn-/Test- from Section “Model Without DBS” is included to facilitate comparison. When DBS is turned ON at test there is no effect of training on either measure but when DBS is turned ON only during training the effect on both measures is in the same direction as those obtained with the control condition (i.e., Learn-/Test-).

Next we examined the effect of DBS on periodicity and striatal synchrony. Given the results shown in Section “Model Without DBS”, it could be expected that periodicity may be related to successful training as evidenced by improvement (or lack thereof) in accuracy. Figure 5 shows the periodicity and striatal synchrony for all conditions, both before and after training (pre- and post-test, respectively). As can be seen, the levels of striatal synchrony and periodicity vary in each condition. There was no effect of training on both periodicity and striatal synchrony when the DBS was ON during testing. Figure 4, shows that the model performed at chance in these two conditions.

In the Learn+/Test- condition, posttest periodicity for incorrect trials was similar to that obtained in the Learn-/Test- condition (and also similar to the Test+ conditions). However, post-test periodicity for correct trials was lower in the Learn+/Test- condition than in the Learn-/Test- condition. In contrast, striatal synchrony decreased after training when the DBS was OFF at test, but there was no difference between correct and incorrect trials. Turning the DBS ON or OFF during the training phase also had no effect. Hence, the difference in post-test periodicity for correct trials may account for the difference in accuracy between the Learn-/Test- and Learn+/Test- conditions (91.1% vs. 73.8%). This suggests that increased periodicity for correct trials may be responsible for the increased accuracy after training.

**Figure 6:**
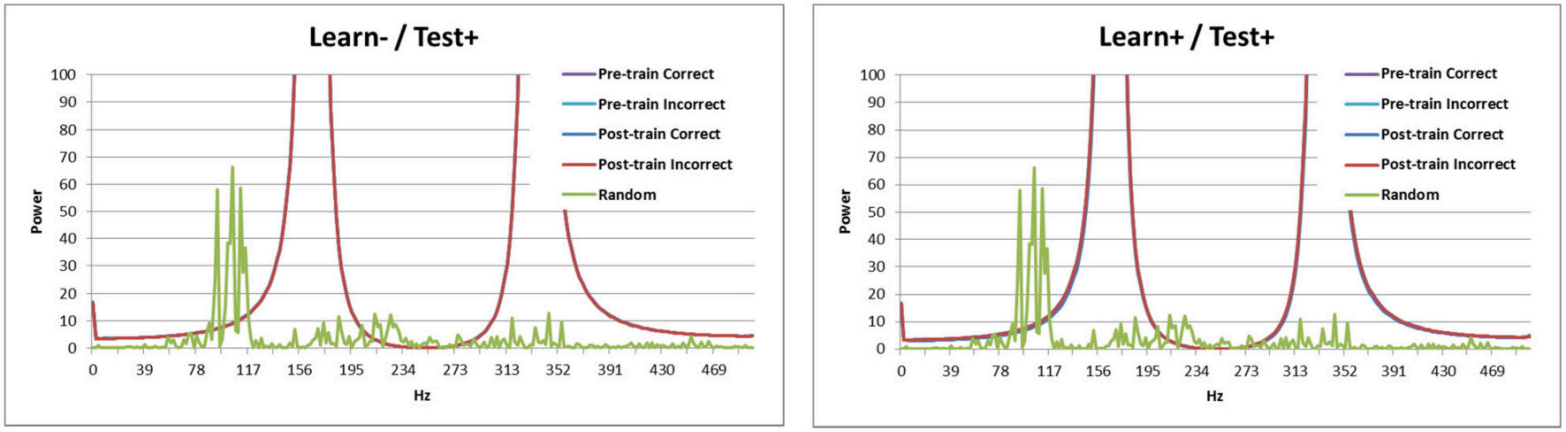
**Spectral decomposition of the two conditions with the DBS ON at test**. As can be seen, the signal is perfectly synced with the fundamental frequency and harmonics of the DBS (all four lines overlap near perfectly). The spectral decomposition for randomly distributed input is also shown for comparison (as described in Figure 3).

**Figure 7:**
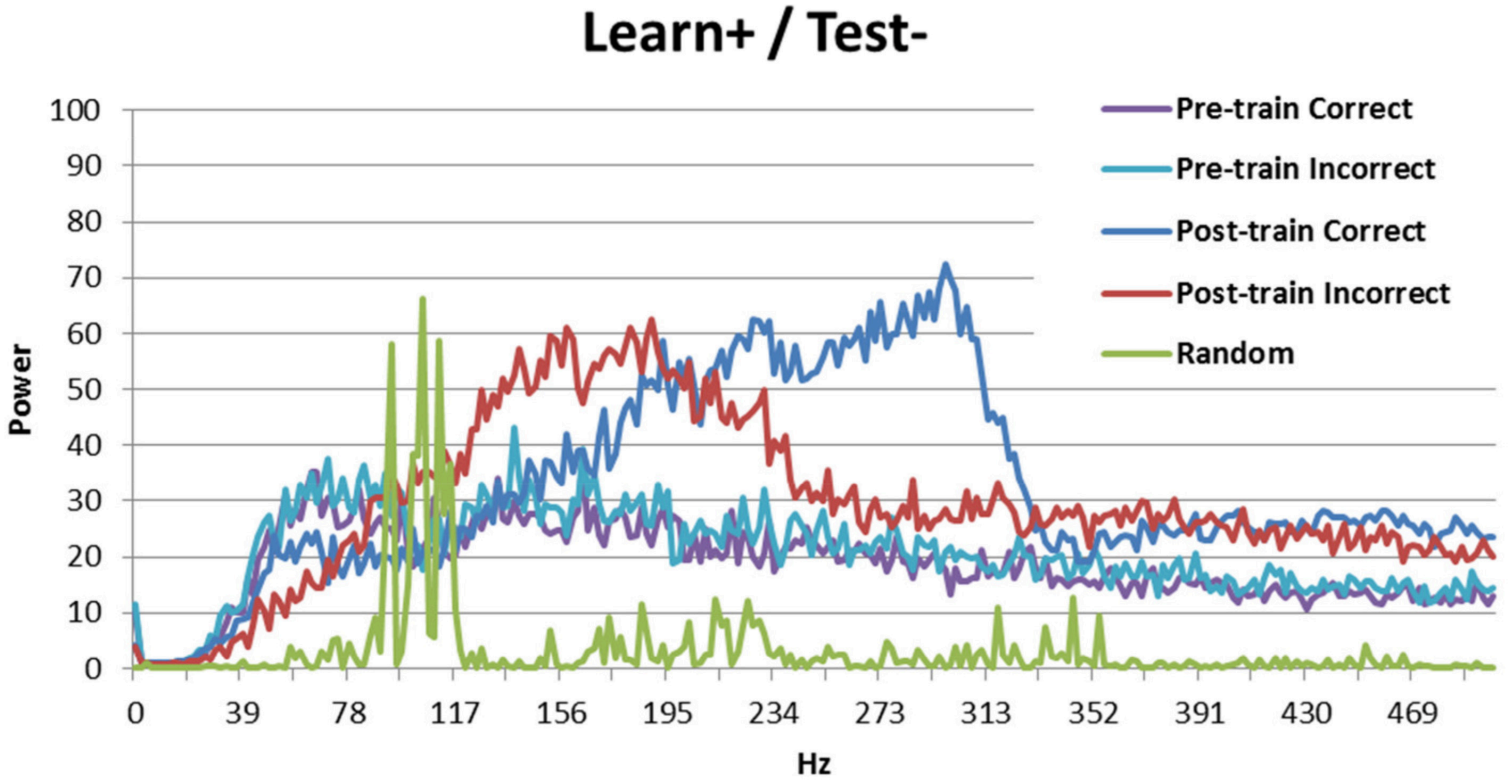
**Spectral decomposition of the Learn+/Test- condition**. As can be seen, the signal is stronger for incorrect trials than for correct trials at moderate frequencies, but there is a crossover and the signal is stronger for correct trials than for incorrect trials in higher frequency bands. The spectral decomposition for random activity is also shown for comparison (as described in Figure 3).

Looking at the spectral decomposition of the three DBS conditions is important for further understanding the model's behavior. These are shown in Figures 6, 7. The spectral decomposition for random activity (denoted in the figure as Random) is also shown for comparison (same as in Figure 3). First, Figure 6 shows the spectral decomposition of the two conditions with the DBS ON at test. As can be seen, the spectral decompositions are identical, and the signal is perfectly synced with the DBS' fundamental frequency (and its harmonics). This shows that the signal from the DBS is regular, and overpowered the cortical stimulation thus making it irrelevant. As a reminder, these two conditions performed at chance.

The Learn+/Test- condition is more interesting, as the model performed better than chance, but not as well as when there was no DBS at learning (Learn-/Test-). Previous analyses suggest that the difference between these two conditions may be periodicity (see Figure 5A). Figure 7 shows the spectral decomposition for the Learn+/Test- condition. As can be seen, the signal is stronger for incorrect trials (red) than for correct trials (blue) in moderate frequency bands, but there is a cross-over and the signal becomes stronger for correct than incorrect trials in higher frequency bands. Comparing these results to those obtained in the Learn-/Test- condition (Figure 3) shows that the strength of the signal is stronger for correct trials than for incorrect trials for a broader frequency band, starting at a lower frequency, in the Learn-/Test- than in the Learn+/Test-. If stronger signal in high frequency bands is related to long-term potentiation [as hypothesized by Brincat and Miller (2015)], then the stronger signal in high frequency should co-occur with correct responses in order for the model to correctly learn the task (the same, but reverse reasoning applies to long-term depression). Because this effect begins lower on the frequency spectrum in the Learn-/Test- condition than in the Learn+/Test- condition, the former condition should learn the task better. This mechanism can account for the higher accuracy in the Learn-/Test- condition.

### Correlation with learning curve

**Figure 8:**
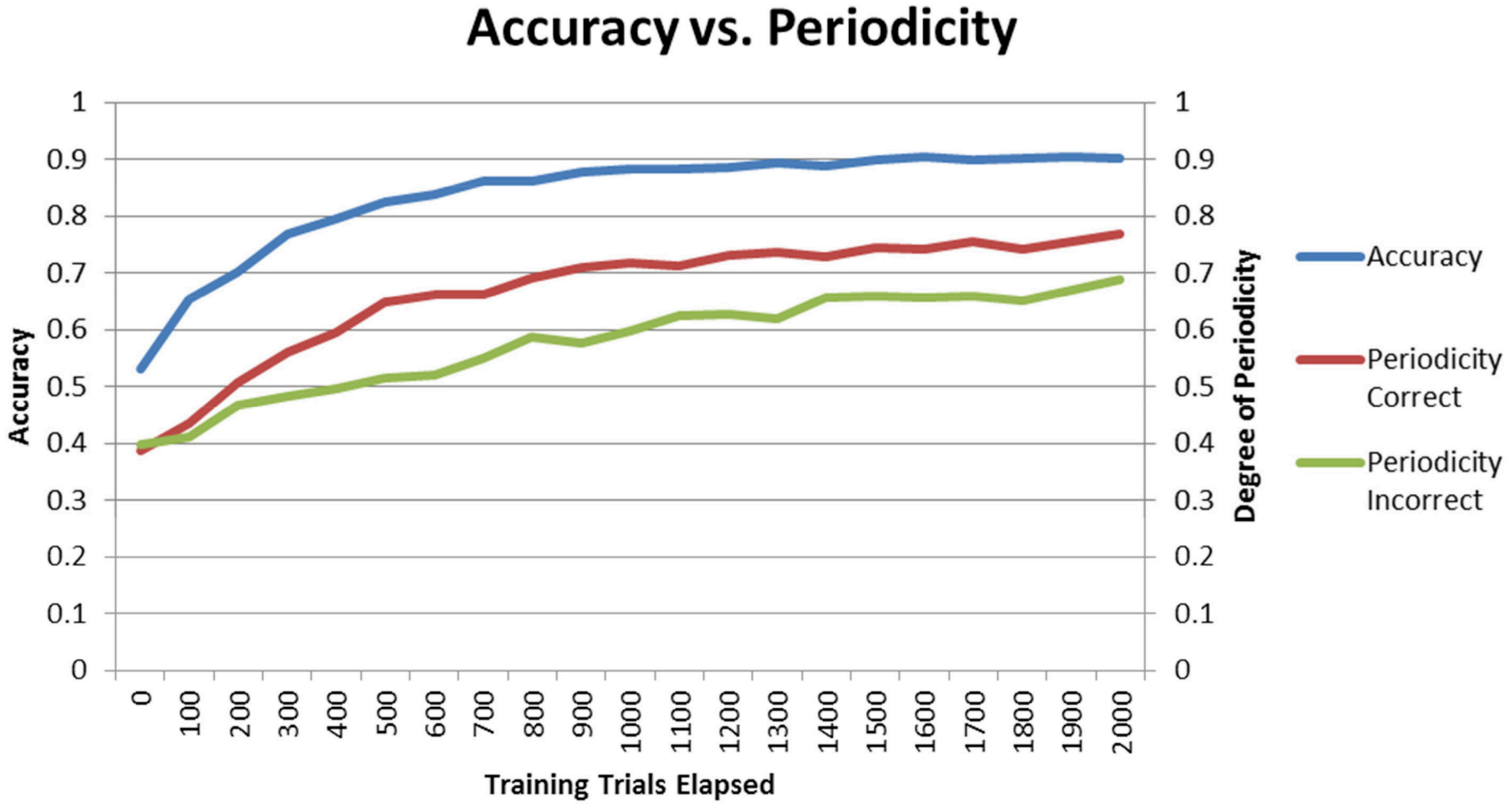
**Accuracy and periodicity as they change with training**. The periodicity of trials where the model responded correctly almost exactly matches the learning curve while the periodicity of trials where the model responded incorrectly is not as correlated with accuracy.

The final performances of the different conditions in Sections “Model Without DBS” and “Model With DBS” suggest that higher periodicity for correct trials is related to higher accuracy. If this is the case, then periodicity for correct trials should increase as the model learns. To test for this hypothesis, the Learn-/Test- condition was re-run with periodicity and accuracy calculated after each block of 100 trials. Figure 8 shows how periodicity and accuracy change over the course of training for correct and incorrect trials. The periodicity of correct trials correlates almost perfectly with accuracy with a correlation coefficient of 0.981. The periodicity of incorrect trials also matches accuracy well with a coefficient of 0.905. However, the correlation between periodicity in correct trials and accuracy is significantly higher than the correlation between periodicity in incorrect trials and accuracy (*Z* = 2.4, *p* < 05). The final accuracy and periodicity for correct trials in Figure 8 below are about 90% and 0.75 (respectively), which reproduces the results earlier obtained in the Learn-/Test- condition in Section “Model Without DBS” (see Figures 4, 5). Furthermore, accuracy reaches 70% after about 200 trials, and the periodicity in correct trials at this point is slightly above 0.5. This corresponds to the final accuracy and periodicity of the Learn+/Test- condition in Section “Model With DBS” (also shown in Figures 4, 5). Together, these results provide strong support for the previous interpretation that periodicity for correct trials is related to accuracy.

## Discussion

This article explored the effect of reinforcement learning on (within-neuron) periodicity and (between-neuron) synchrony in the striatum. This was accomplished by adding cortical input and a reinforcement learning algorithm to the Ponzi and Wickens (2010) striatal model. The new model was used to simulate a simple instrumental conditioning task. Neural periodicity and striatal synchrony was disrupted by simulating DBS (Rubin and Terman, 2004) either during training and/or at test. The results show that (1) periodicity increases with training and (2) striatal synchrony decreases with training (but this may be an artifact linked to increases in firing rates). In addition, (3) there is a highly positive correlations between accuracy and periodicity in correct trials. This correlation is stronger for correct responses than for incorrect responses. Finally, (4) successful learning is accompanied by a stronger signal for correct trials than incorrect trials in high frequency bands. Below we explore some implications of these results.

### Striatal synchrony

Striatal synchrony was reduced after the model had learned. This suggests that one of the effects of reinforcement learning is to decrease striatal synchrony. However, applying the DBS increases the striatal synchrony and returns the performance to chance. As explained in Section “Introduction”, abnormal synchrony between different brain areas, such as the globus pallidus and the subthalamic nucleus, is related to some cognitive deficits (e.g., as in Parkinson's disease: Bergman et al., 2010). However, the simulation results suggest that low striatal synchrony does not cause accurate performance in an instrumental conditioning task. First, the striatal synchrony is similar for the Learn-/Test- and the Learn+/Test- conditions, and yet their accuracy differs. Second, the striatal synchrony is similar for correct and incorrect trials. In other words, different accuracies do not imply different synchronies, and the same level of synchrony is measured in correct and incorrect trials. Third, simulating random spiking times with the pre- and post-test firing rates produces synchrony similar to that observed in the model. Together, these results suggest that the striatal synchrony is a byproduct of reinforcement learning, possibly related to the higher neural activity in the inhibitory network, but is not responsible for the higher accuracy following training.

### Neural periodicity

The correlation between accuracy and periodicity is high, and even more so in correct trials. As training progresses, both the accuracy and the periodicity increase. This suggests that periodicity in correct trials may be related to response accuracy in the network. As a case in point, Figure 8 shows that the final performance of the Learn+/Test- condition constitutes an intermediate stage of learning in the Learn-/Test- condition (with similar accuracy and periodicity for correct trials). However, like for striatal synchrony, periodicity does not appear to be sufficient for accuracy. For example, turning ON the DBS produces relatively high periodicity, higher than in pretest when the DBS is OFF, and yet the model performs at chance. However, every condition that we tested that resulted in improved accuracy also resulted in increased periodicity, and the fact that periodicity is higher for correct than incorrect trials suggest that high periodicity may be necessary for network accuracy. Periodicity may facilitate communication between brain areas because it makes neural firing more predictable.

### Spectral decomposition

The spectral decomposition provided the most interesting results. First, when the DBS is ON, the neural activity perfectly synchronize with the DBS' fundamental frequency, thus making cortical stimulation irrelevant. This result clearly illustrates why the model performs at chance when the DBS is ON. When the DBS is OFF, the signal is stronger in correct trials than in incorrect trials in high frequency bands. This is in line with observations in monkeys by Brincat and Miller (2015), and may be causing long-term potentiation following correct responses and long-term depression following incorrect responses. When the DBS was ON at training (Learn+/Test-), this phenomenon is only observed for a much higher frequency band than when the DBS was OFF the whole time (Learn-/Test-), which can explain the difference in accuracy. Overall, stronger signal for correct trials (and weaker signal in incorrect trials) in high frequency domains may be key to successful learning.

### Limitations and future work

The present work suggests that simulating network dynamics may allow for exploring the relationship between cognitive functions and brain rhythms. However, the simulated network is still “small scale” when compared to realistic brain networks, and does not represent the full network required for instrumental conditioning (Helie et al., 2013). Hence, it is likely that some of the effects observed would be modified by the rest of the circuit. Future work should focus on simulating a more complete network. In addition, synchrony, periodicity, and spectral decompositions are only three measures of the dynamics of the network. Future work should use other measures of network dynamics to explore their relationship to accuracy in instrumental conditioning (and possibly classical conditioning) in other cognitive tasks. Finally, the DBS model was modified to produce a net current of zero, which makes the DBS less biologically realistic. This was justified because its role in the current work was limited to disrupting the striatal rhythms. Future work could use a more realistic DBS model, that only affects part of the striatum, and explore the effect of realistic DBS stimulation on striatal activity.

## Author contributions

SH designed the experiment and hypotheses, and interpreted the results; PF wrote the code, performed the simulations and analyses; The manuscript was written by both authors.

### Conflict of interest statement

The authors declare that the research was conducted in the absence of any commercial or financial relationships that could be construed as a potential conflict of interest.
