# Supplementary material for: Simulating the Effect of Reinforcement Learning on Neuronal Synchrony and Periodicity in the Striatum
Source: Front Comput Neurosci. 2016 Apr 29;10:40. doi: 10.3389/fncom.2016.00040 (PMC4850239; doi:10.3389/fncom.2016.00040)
Supplement: Supplementary file 1 [file DataSheet1.docx]

# Appendix

The Ponzi and Wickens (2010) model is a randomly-connected single layer model of sparse lateral inhibition. The striatal neurons are modeled using the *I*_Na,p_ + *I*_k_ model from Izhikevich (2006):

$$C\frac{dV_{i}}{dt}=I_{i}\left( t \right)-J_{i}(t)-g_{L}\left( V_{i}-E_{L} \right)-g_{\mathrm{Na}}{(m}_{\infty}(V_{i}))\left( V_{i}-E_{\mathrm{Na}} \right)-g_{K}n_{i}(V_{i}-E_{K})$$

$$\frac{dn_{i}}{dt}=(n_{\infty}\left( V_{i} \right)-n_{i})/\tau_{n}$$

$$x_{\infty}\left( V \right)=\frac{1}{1+\exp\left( \frac{V_{\infty}^{x}-V}{k_{\infty}^{x}} \right)}$$

where C = 1 is the membrane capacitance, *V_i_* is the membrane potential of neuron *i*, *t* is time in milliseconds, *I_i_(t)* is the excitatory input to neuron *i* at time *t*, and *J_i_(t)* is the inhibitory input to neuron *i* at time *t*. g_L_ = 8 is the leak conductance, E_L_ = -80 is the leak equilibrium potential (also known as the reversal potential), and g_Na_ = 20 and E_Na_ = 60 are the sodium conductance and equilibrium potential respectively. Likewise, g_K_ = 10 and E_K_ = -90 are respectively the potassium conductance and equilibrium potential. Each g(*V -* E) term models the flow of a specific ion across the cell membrane; in order: leak (mostly Cl^-^), Na^+^, and K^+^ ions. *n_i_* models the slow behavior of the K^+^ current.

*m_∞_(V)* and *n_∞_(V)* are defined by the equation for *x_∞_(V)* above, substituting *m* or *n* for each *x* in the equation. These model the response of the various ion channels to changes in cell membrane potential. K^+^ channels respond slowly and thus *n_∞_(V)* is embedded in *n* while Na^+^ channels respond quickly and can be modeled as responding instantly. Hence, *m_∞_(V)* is directly used in the membrane potential equation. τ_n_ is set to 1. V_∞_^m^ = -20 and V_∞_^n^ = -25 are the values such that *x_∞_*(V_∞_^x^) = 0.5. k_∞_^m^ = 15 and k_∞_^n^ = 5 are the slope factors. V_syn_ is the cell resting potential of -65.

Inhibitory lateral connections within the striatum were set randomly. For every pair of neurons A and B (A≠B), there is a 0.2 probability that A sends inhibitory signals to B. The strength of the signal *g_i_* sent by striatal neuron *i* before being modified by the connection strength is calculated by the following:

$$\tau_{g}\frac{dg_{i}}{dt}=H\left( V_{i}\left( t \right)-V_{\mathrm{th}} \right)H\left( dV_{i}\left( t \right) \right)H\left( -dV_{i}(t-1) \right)-g_{i}(t)$$

Here V_th_ is the spiking threshold (a membrane potential above this will result in a spike even in the absence of any further input) and H() is the Heaviside function. The Heaviside functions are multiplied together and thus act like binary ‘*And’* operators. τ_g_ is set to 50. Before this signal is received by any post-synaptic striatal neurons it is modified by a weight unique to each connection. The weights are chosen at the start of the simulation using the following method:

$$W_{ij}=r_{ij}\left( \frac{k_{syn}}{s*p} \right)$$

where *W_ij_* is the weight of the connection from neuron *j* to neuron *i* and *K_syn_* is 85. *r_ij_* is a random number unique to the connection between *i* and *j* and is uniformly distributed from 0.8 to 1.2. The weights are divided by the size of the striatal network (*s*) and the network connectivity (*p*) so that on average each neuron receives the same amount of inhibition regardless of network size or connectivity. Thus the formula for the inhibitory input to neuron *i* is:

$$J_{i}\left( t \right)= \sum_{j} g_{i}(t)W_{ij}\left( V_{i}(t)-V_{\mathrm{syn}} \right)$$

where all terms are as defined above.
